# Supplementary figures and images for: Synchrotron Radiation Refraction-Contrast Computed Tomography Based on X-ray Dark-Field Imaging Optics of Pulmonary Malignancy: Comparison with Pathologic Examination
Source: Cancers (Basel). 2024 Feb 16;16(4):806. doi: 10.3390/cancers16040806 (PMC10886596; doi:10.3390/cancers16040806)

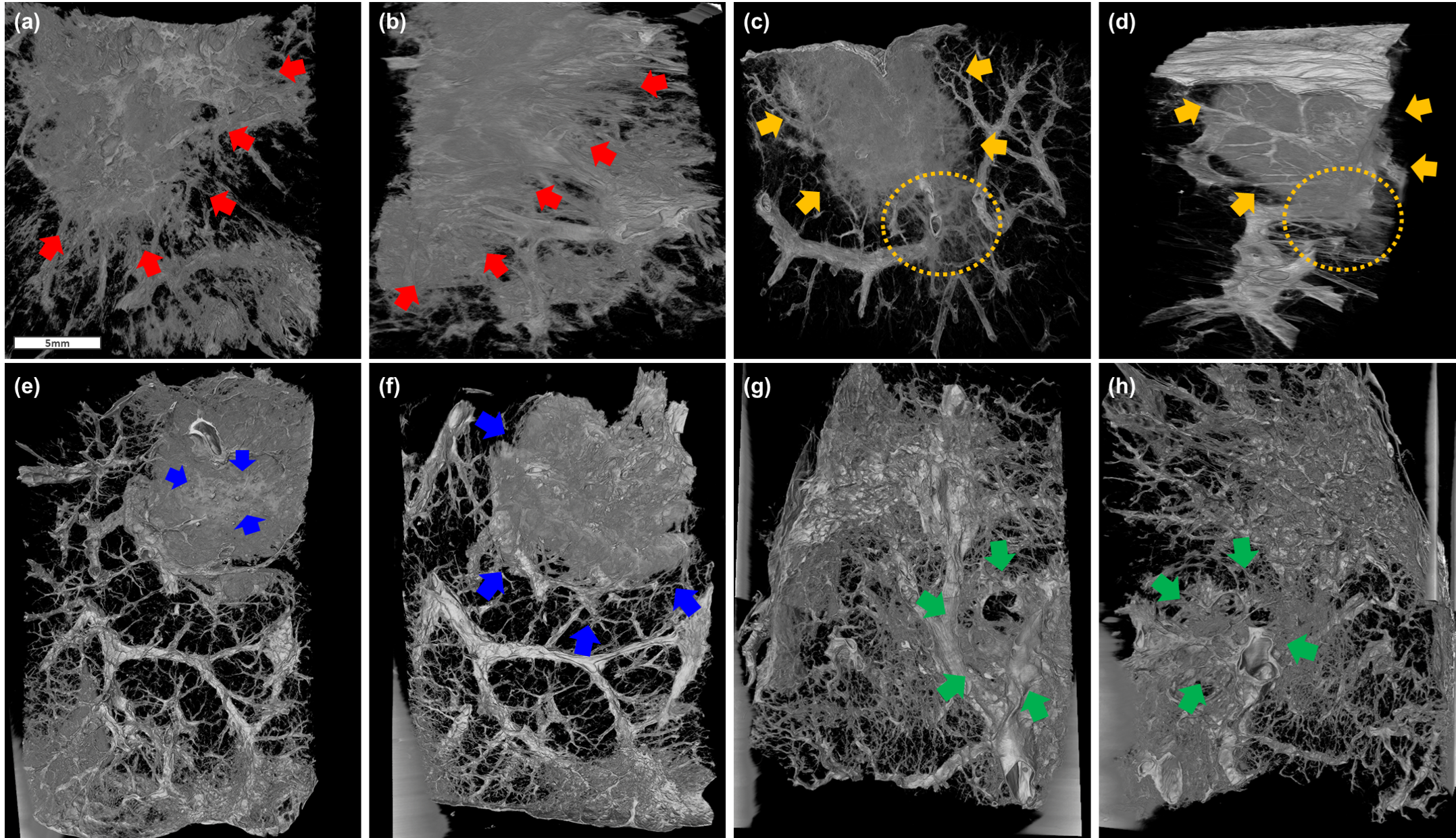

Supplement: Supplementary file 1 [file cancers-16-00806-s001.zip › Figure S1.png]
